# Characterization of anoikis-based molecular heterogeneity in pancreatic cancer and pancreatic neuroendocrine tumor and its association with tumor immune microenvironment and metabolic remodeling

**DOI:** 10.3389/fendo.2023.1153909

**Published:** 2023-05-10

**Authors:** Ning Li, Xingqing Jia, Zhong Wang, Kaige Wang, Zumin Qu, Dong Chi, Zhubo Sun, Jian Jiang, Yougang Cui, Changmiao Wang

**Affiliations:** ^1^ Department of General Surgery, First Affiliated Hospital of Dalian Medical University, Dalian, Liaoning, China; ^2^ Department of General Surgery, Wafangdian Central Hospital, Dalian, Liaoning, China; ^3^ Graduate School of Dalian Medical University, Dalian, Liaoning, China; ^4^ Department of Digestive, Jinan City People’s Hospital, Jinan, Shandong, China; ^5^ Department of Pathology, Wafangdian Central Hospital, Dalian, Liaoning, China; ^6^ Department of General Surgery, The Affiliated Zhongshan Hospital of Dalian University, Dalian, Liaoning, China

**Keywords:** pancreatic adenocarcinoma, pancreatic neuroendocrine tumors, anoikis, molecular characteristics, metabolic remodelling, tumor immune microenvironment

## Abstract

**Background:**

Accumulating evidence suggests that anoikis plays a crucial role in the onset and progression of pancreatic cancer (PC) and pancreatic neuroendocrine tumors (PNETs); nevertheless, the prognostic value and molecular characteristics of anoikis in cancers are yet to be determined.

**Materials and methods:**

We gathered and collated the multi-omics data of several human malignancies using the TCGA pan-cancer cohorts. We thoroughly investigated the genomics and transcriptomics features of anoikis in pan-cancer. We then categorized a total of 930 patients with PC and 226 patients with PNETs into distinct clusters based on the anoikis scores computed through single-sample gene set enrichment analysis. We then delved deeper into the variations in drug sensitivity and immunological microenvironment between the various clusters. We constructed and validated a prognostic model founded on anoikis-related genes (ARGs). Finally, we conducted PCR experiments to explore and verify the expression levels of the model genes.

**Results:**

Initially, we identified 40 differentially expressed anoikis-related genes (DE-ARGs) between pancreatic cancer (PC) and adjacent normal tissues based on the TCGA, GSE28735, and GSE62452 datasets. We systematically explored the pan-cancer landscape of DE-ARGs. Most DE-ARGs also displayed differential expression trends in various tumors, which were strongly linked to favorable or unfavorable prognoses of patients with cancer, especially PC. Cluster analysis successfully identified three anoikis-associated subtypes for PC patients and two anoikis-associated subtypes for PNETs patients. The C1 subtype of PC patients showed a higher anoikis score, poorer prognosis, elevated expression of oncogenes, and lower level of immune cell infiltration, whereas the C2 subtype of PC patients had the exact opposite characteristics. We developed and validated a novel and accurate prognostic model for PC patients based on the expression traits of 13 DE-ARGs. In both training and test cohorts, the low-risk subpopulations had significantly longer overall survival than the high-risk subpopulations. Dysregulation of the tumor immune microenvironment could be responsible for the differences in clinical outcomes between low- and high-risk groups.

**Conclusions:**

These findings provide fresh insights into the significance of anoikis in PC and PNETs. The identification of subtypes and construction of models have accelerated the progress of precision oncology.

## Introduction

Globally, pancreatic cancer (PC) is regarded as a lethal gastrointestinal malignancy with a mortality rate proportional to its occurrence (1, 2). Exposure to risk factors such obesity, diabetes, cigarette use, and alcohol intake is connected to the poor prognosis and steady incidence rates of PC patients; however, early-stage nonspecific symptoms also contribute to the diagnosis (3). In addition to pancreatic cancer, neuroendocrine tumors are also relatively common types of pancreatic tumors. The origin of pancreatic neuroendocrine tumors (PNETs) is concealed, and their biological activity is highly variable, being characterized by passive growth, invasive development, and even early metastasis; their biological characteristics may change as the disease progresses. As a result of the tumor’s function in hormone release, PNETs may produce hormone-related symptoms or syndromes, and there are significant differences in prognosis between PNETs of different grades and stages. In modern medicine, surgery remains the therapeutic cornerstone of PC and PNETs, complemented by other, more all-encompassing treatments like radiation and chemotherapy (4). Despite extensive therapy, PC has a dismal 5-year survival rate of around 7% at present (4). The urgent need to establish the potential heterogeneity of PC and PNET patients is a necessary step in addressing this issue. This would enable physicians to generate more accurate prognoses on patient outcomes and swiftly execute tailored treatment programs.

Anoikis was initially identified in 1994, indicating that normal adhering cells would die of “homelessness” if they were suspended for an extended period of time (5). Anoikis is a kind of programmed cell death and is associated with “suicidal” cell activity (5). It is caused by separation from extracellular matrix (6). It is essential for maintaining the integrity of the body’s tissues, and its primary role is to inhibit improper cell proliferation or attachment to abnormal extracellular matrix (7). Loss of nested apoptosis resistance is the basis of tumor spread, metastasis, and invasion, since it enables tumor cells to migrate to distant new tissues or lymph nodes through lymphatic or blood circulation and continue to grow (8). Loss of tumor cells and resistance to apoptosis play a significant role in the invasion and metastasis of pancreatic cancer.

In this study, we systemically summarized the pan-cancer landscape of anoikis for the first time. Based on the anoikis scores, 930 patients with PC were precisely stratified into three subtypes accompanied by distinct prognoses and tumor immune microenvironment. These three subtypes included anoikis-active, anoikis-normal, anoikis-inactive subpopulations. The patients in anoikis-active subtype had higher anoikis scores and worse prognoses, indicating the carcinogenic effects of anoikis in PC. 226 patients with PNETs were also stratified into S1 and S2 subtypes with distinct molecular characteristics. Finally, we also developed a novel anoikis-based prognostic model for patients with PC, which help promote the development of oncology precision.

## Methods

### Data collection and processing

A total of 794 anoikis-related genes (ARGs) were downloaded from the GeneCard website (https://www.genecards.org/). Among them, 501 ARGs with gene scores > 0.4 were preserved for further analysis (**Table S1**). Pan-cancer cohorts including gene expression profiles, mutation information, methylation levels, and clinical data were obtained from the Firehose (http://gdac.broadinstitute.org) and Xena Browser (https://xenabrowser.net/datapages/) platforms (9). A total of 930 PC and 171 para-cancerous tissues’ transcriptomics data and their corresponding clinical data were acquired from the publicly free platforms, including ArrayExpress (https://www.ebi.ac.uk/arrayexpress), The Cancer Genome Atlas (TCGA, https://portal.gdc.cancer.gov), International Cancer Genome Consortium (ICGC, https://dcc.icgc.org/), Gene Expression Omnibus (GEO, https://www.ncbi.nlm.nih.gov/geo/), and Genotype-Tissue Expression (GTEx) databases (10–14). Of note, patients without follow-up information were excluded in this study.

In addition to these, the transcriptomics data of 226 patients with PNETs were also collected and complied from the public websites. The ICGC-PAEN-AU cohort provided the data of 32 PNETs samples, GSE98894 cohort (15) provided the data of 113 PNETs samples, GSE73338 cohort (16) provided the data of 81 PNETs samples. A total of 171 normal pancreas samples consisting of 4 samples from TCGA platform and 167 samples from GTEx website were also collected as the control group. In order to eliminate the batch effects derived from the different platforms, a well-recognized bioinformatics algorithm, called ComBat, was utilized. The ComBat function was developed on the basis of the “sva” package in R (17).

The overall analysis strategies of this research are summarized as follows: a) To filter ARGs significantly associated with the occurrence of PC, the differentially expressed ARGs (DE-ARGs) were determined with the help of limma package in R. In the process of above analysis, three cohorts including GSE28735 (18, 19) (45 tumor samples vs 45 normal samples), GSE62452 (20) (69 tumor samples vs 61 normal samples), and TCGA+GTEx cohorts (178 tumor samples vs 171 normal samples) were utilized, and DE-ARGs were identified by taking the intersection of the results of above three cohorts. Subsequently, pan-cancer analysis highlighted the pivotal contributions of DE-ARGs in multiple human cancers. The specific analytic methods were similar to the previous studies (21, 22). b) The single sample gene set enrichment analysis (ssGSEA) was performed to evaluate the relative activities of 930 PC patients’ anoikis signaling pathway. Cluster analysis was then carried out to classify 930 PC patients into three distinct subtypes with different anoikis activities. c) Considering the limitations for the clinical application of cluster results, a novel anoikis-related prognostic model (ARPM) was developed and validated. We separated 930 PC patients into two cohorts (i.e. training dataset and validation dataset). Among them, GSE57495 (23), GSE28735, GSE62452, E-MTAB-6134 (24), and TCGA-PC datasets including a total of 635 PC samples were compiled as a training cohort for future research, while 295 PC patients in the ICGC-CA and ICGC-AU datasets were defined as a validation cohort. d) 226 patients with PNETs were also performed cluster analysis to determine the possible heterogeneity.

### Cluster analysis based on anoikis activities

Using single-sample gene set enrichment analysis (ssGSEA), enrichment scores for the anoikis pathway in patients with PC and PNETs were calculated. The “Gplots” and “pheatmap” R packages were used to display heatmaps incorporating DE-ARGs expression, anoikis scores, and clinical clusters for both PC and PNETs. Brown indicated that the expression of the gene was larger in tumor samples than in normal samples, while dark blue indicated the opposite. The status of mRNA expression in tumor tissues was categorized into 3 clusters: high expression of the ARGs, normal expression of the ARGs, and low expression of the ARGs. The violin plots were depicted to compare the anoikis enrichment scores between distinct clusters. Of note, those clusters with similar enrichment scores were further consolidated into one cluster. Higher scores indicated increased DE-ARG expression levels, whereas lower scores indicated the reverse. More importantly, we also evaluated the disparities in the distribution of immunological and metabolic pathways among diverse clusters.

### Drug sensitivity analysis for PC patients

The R package “pRRophetic” was used to predict chemotherapy response in order to better comprehend the relationship between anoikis pathway gene expression and malignancy medication treatment. As one of the largest public repositories of information on cancer drug sensitivity, drug responses, and molecular targets, the “pRRophetic” package, which was based on the Cancer Genome Project (CGP) and contained 138 anticancer drugs against 727 cell lines, allowed for the identification of novel therapeutic targets to improve cancer treatment (25). Meanwhile, the semi-maximum inhibitory concentration (IC50) of the samples was calculated using the ridge regression approach. A smaller IC50 was usually related to a lower semi-inhibitory mass concentration of the drug in cancer cells, suggesting that the cancer cells were more vulnerable to the medication.

### Associations of the anoikis scores with the classical cancer-related genes and tumor immune microenvironment in PC patients

The fundamental unit of genetic information is the gene. In general, two types of genes (i.e. oncogenes and tumor suppressor genes) in cells are intimately associated with the emergence and growth of tumors. Oncogenes are usually genes with the functions of promoting cell growth, activating cell cycle and inhibiting the level of apoptosis. Tumor suppressor genes negatively control cell development and cell cycle, induce apoptosis, and repair DNA damage. Considering the important role of oncogenes and tumor suppressor genes in tumorigenesis, we further analyzed the correlation between anoikis scores and these genes. Using the “pheatmap” and “gplots” packages in RStudio, we produced a heatmap showing the expression levels of various oncogenes and tumor suppressor genes in the three clusters in order to explore the likely regulatory mechanism of the anoikis pathway in PC.

The tumor immune microenvironments of three PC subtypes were then compared. We intensively examined the algorithms MCPCOUNTER, XCELL, CIBERSORT, EPIC, CIBERSORT-ABS, and TIMER for assessing cell immune responses or cellular components across the three subtypes of PC. Several algorithms were used to plot a heatmap to identify shifts in immune response. Immune checkpoint functioned as the major manager of immune cell activity. Thus, we also investigated the expression features of immune checkpoint-related genes among various clusters.

In addition, the 29 well-recognized immune-associated gene sets were also quantified for assessing the scores of immune cells and immune-related functions using ssGSEA (26). The scores of immune cells and immune-related functions might partially represent the quantity of immune cell infiltration and the activation of immunological-related processes. Subsequently, the Spearman correlation analysis was employed to explore the correlation between ARG scores and immune scores. We created a scatter plot using the “ggscatterstats” package to show the relationships between the four immune-infiltrating components (macrophages, parainflammation, TIL, and Th1 cells) and the anoikis pathway scores. Finally, using Spearman’s correlation coefficient, the R Studio tools “ggplot2” and “dplyr” were then applied to generate a heatmap illustrating the relationship between ARGs and immune cell infiltration (ICI).

### Development and validation of a prognostic signature based on DE-ARGs

As a further step, we performed LASSO regression analysis on 40 DE-ARGs, with the minimal criteria determining the penalty parameter (λ). $Risk\;score\;=\sum_{k=1}^{n}expk\;*\;\beta k$ . Using the median risk score, 930 individuals with PC from the training and validation cohorts were classified into high- and low-risk categories. The training cohort involved 635 PC patients from GSE57495, GSE28735, GSE62452, MTAB-6134, and TCGA-PC datasets, whereas, the validation cohort involved 295 PC patients from ICGC-CA and ICGC-AU datasets. For both training and validation cohorts, survival analyses using the KM technique were carried out to determine whether the signature could be used to forecast survival. ROC curves of 1-, 3-, 5-, and 7-years were also plotted to quantitatively evaluate the predictive ability of our prognostic model.

### Immune cell infiltration and immune checkpoint gene expression differences between low-risk and high-risk subgroups

Based on the previous results of ICI assessment, the heatmaps were utilized to show the discrepancies in the tumor immune microenvironment between low- and high-risk subgroups. Each color represented different ICI prediction algorithms. The differential expression of common immune checkpoint genes (ICGs) in high-risk and low-risk categories was also examined, with only statistically significant results (p< 0.05) being displayed. The above analysis is performed in both the train and test cohorts.

### Clinical significances of model genes in PC

We integrated the prognosis information, clinical stage, and expression of model genes to highlight their clinical significances. Both univariate Cox regression analysis and Kaplain-Meier analysis were employed to explore and validate their prognostic values. The GEPIA2 platform (http://gepia2.cancer-pku.cn/#analysis) was implemented to analyze their association with clinical stages. The BEST platform (https://rookieutopia.com/app_direct/BEST/#PageHomeAnalysisModuleSelection) was utilized to explore the expression traits of ARGs with clinical significances. Only DE-ARGs with prognostic significances and stage correlation were considered to be closely related to the occurrence and progression of PC.

### Quantitative real-time PCR, immunohistochemistry and immunofluorescence

The MiaPaca-2 cell line was procured from BeNa Culture Collection, and Procell Life Science & Technology Co., Ltd. supplied the HPDE6-C7, CF-PAC1, Panc-1, and BxPC-3 cell lines. DMEM mixed with 10% FBS (Gibco, USA) was utilized to culture HPDE6-C7 (a human pancreatic ductal epithelium cell line), MiaPaca-2, and Panc-1 cell lines, while IMDM mixed with 10% FBS (Procell, China) was used for CF-PAC1, and BxPC-3 was cultured with 1640 mixed with 10% FBS (Procell, China). All the cell lines were incubated in a cell incubator maintained at a temperature of 37°C and with a CO2 concentration of 5%.

By using TRIzol extraction tool provided by Accurate Biotechnology, mRNAs associated with five different cell lines were isolated. These mRNAs were then reversed transcribed into cDNAs using the Reverse Transcription Reagent. The RT-PCR was executed by utilizing the qPCR Kit from Accurate Biotechnology. All reagents used in the experiment were provided by our laboratory. β-actin was selected as the control standard for the experiment, and the mRNA expression level analysis was calculated using the ΔΔCt method. The primer sequences were synthesized from Sango Biotech (Shanghai, China) shown as follows: HK2: 5’- TCCCCTCTCGCGTCTCC-3’(F), 5’- AGAGATACTGGTCAACCTTCTGC-3’(R); MMP11, 5’- GATCGACTTCGCCAGGTACT -3’(F), 5’- CCCCGATAGTCCAGGTCTCA-3’(R); CDH3, 5’- GACACCCATGTACCGTCCTC-3’(F), 5’- TCTCTCCCCTCCCCTCAATTA-3’(R); PDK4, 5’- CCAAGCCACATTGGAAGCAT-3’(F), 5’- TGAACACTCAAAGGCATCTTGG-3’(R); SERPINB5, 5’- ATGCCAAGGTCAAACTCTCCATTCC-3’(F), 5’- CAGCCCTAGATTTTCCAGACAAGCC-3’(R); SLC2A1, 5’- TGGCATCAACGCTGTCTTCT-3’(F), 5’- AGCCAATGGTGGCATACACA-3’(R); β-actin: 5’-CCTGGGCATGGAGTCCTGTG-3’(F), 5’- TCTTCATTGTGCTGGGTGCC-3’(R).

Ultimately, the HPA platform was employed to investigate the protein levels and cellular location of model genes in PC through immunohistochemistry and immunofluorescence techniques.

## Results

### Identification of DE-ARGs between tumor and normal tissues

**Figure 1:**
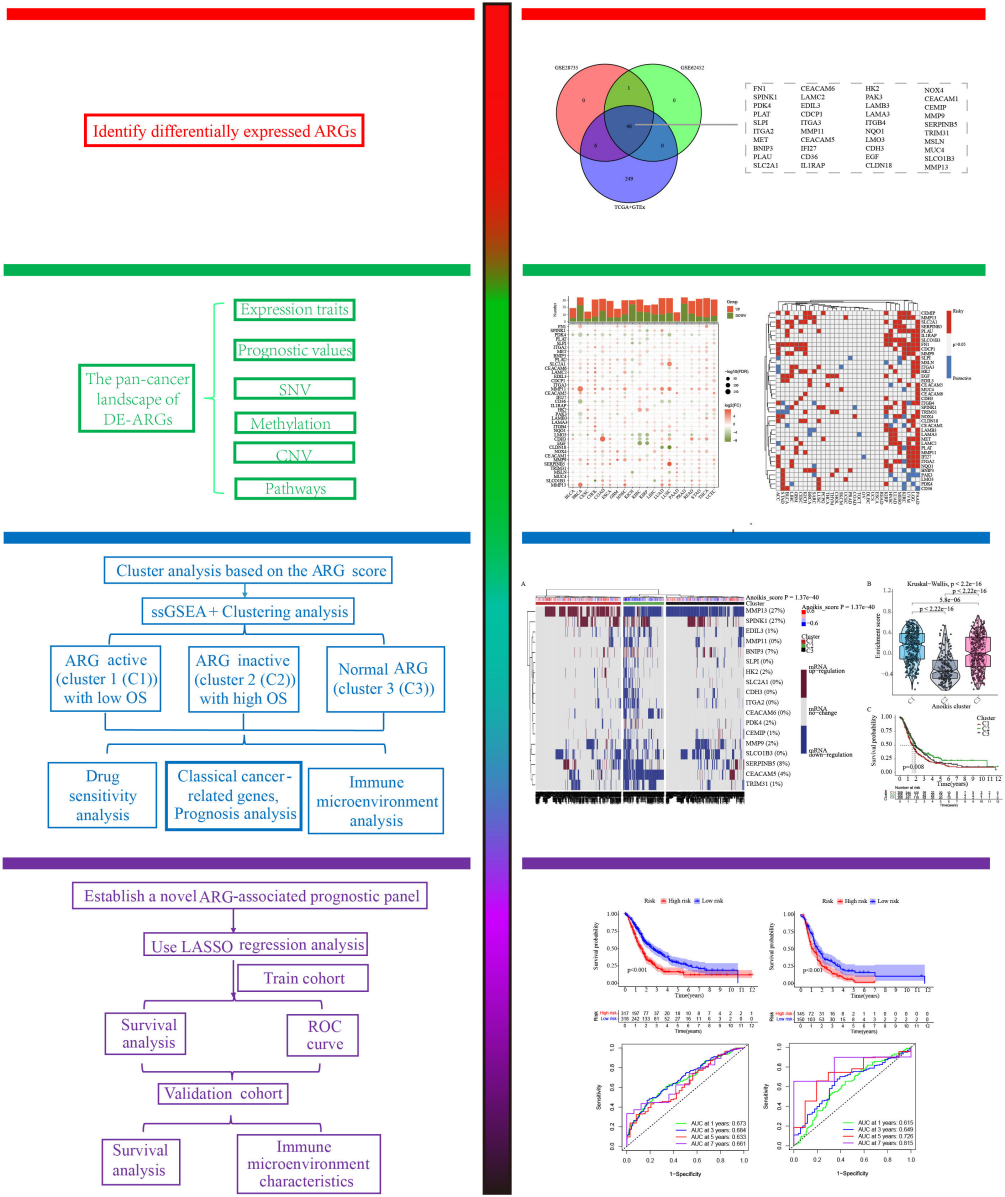
The study-flow of this research.

**Figure 2:**
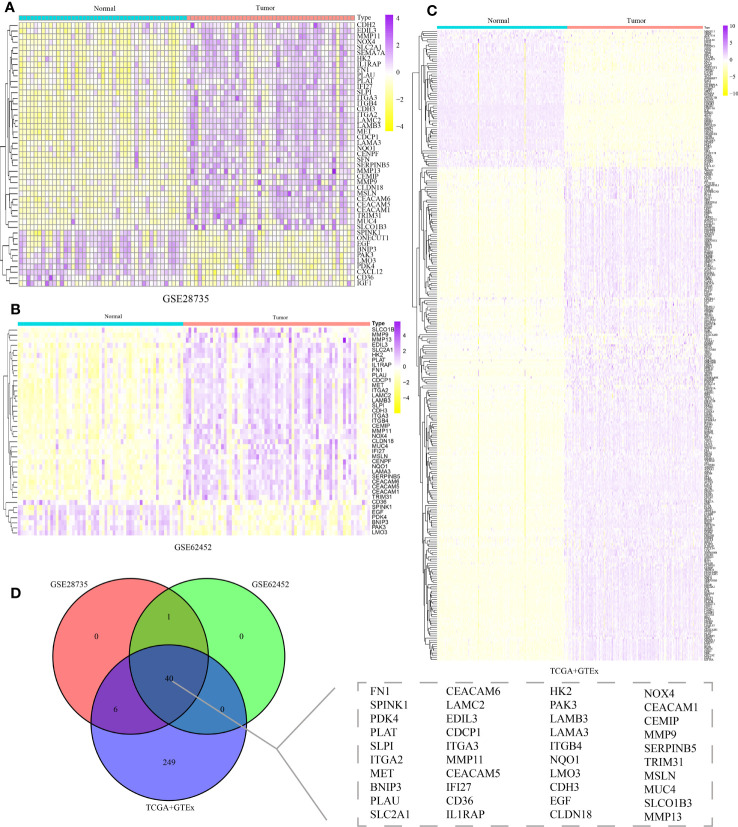
Identification of differentially expressed anoikis-related genes (DE-ARGs). Differential expression analysis of DE-ARGs in **(A)** GSE28735, **(B)** GSE62452, and **(C)** TCGA+GTEx cohorts. **(D)** Identification of 40 shared DE-ARGs.

The workflow of this study was displayed in **Figure 1**. In order to explore the ARGs closely associated with occurrence of PC, the differential expression analyses of three public cohorts were carried out through the limma package in R. The results of GSE28735, GSE62452, and TCGA+GTEx cohorts identified 47, 41, and 295 DE-ARGs, respectively, which were further visualized *via* the heatmap package (**Figures 2A–C**). After taking the intersection of three cohorts, a total of 40 shared DE-ARGs were determined for further analysis (**Figure 2D**; **Table S2**).

### Pan-cancer analysis characterization of the important roles of DE-ARGs

**Figure 3:**
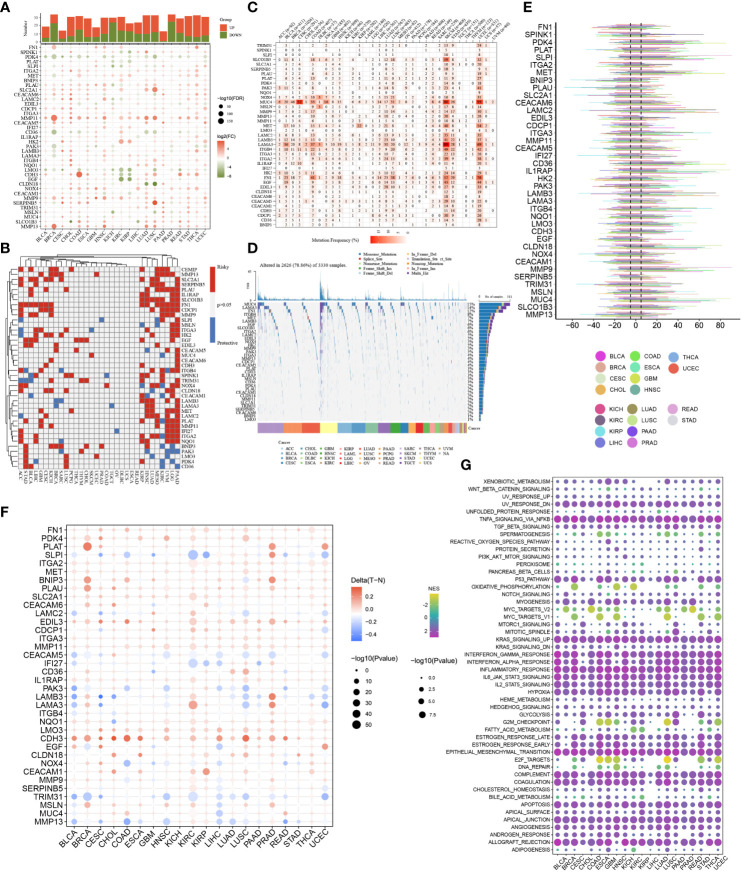
Pan-cancer overview of DE-ARGs. **(A)** mRNA expression traits of DE-ARGs in pan-cancer. **(B)** Prognostic values of DE-ARGs in pan-cancer. **(C, D)** SNV traits of DE-ARGs in pan-cancer. **(E)** CNV traits of DE-ARGs in pan-cancer. **(F)** Methylation levels of DE-ARGs in pan-cancer. **(G)** Pathway regulation ability of DE-ARGs in pan-cancer.

Up to now, the potential roles of DE-ARGs in the occurrence and progression of human multiple cancers remained unclear. Thus, we systematically summarized their pan-cancer characteristics through a series of complex bioinformatics algorithms. Interestingly, differentially expressed genes in pancreatic cancer and para-cancerous tissues showed a similar expression trend in other malignant tumors (**Figure 3A**). The expression levels of SLC2A1, MMP11, HK2, MMP7, and MMP13 in most tumors were significantly increased compared to corresponding para-cancerous tissues, suggesting their potential carcinogenesis. The expression levels of PDK4, LMO3 and PAK3 in most tumors were significantly decreased compared to corresponding para-cancerous tissues, suggesting their potentially protective roles. More importantly, nearly all the DE-ARGs exerted the pivotal parts in the clinical outcomes of patients with PAAD, LGG, UVM, and KIRC, which further highlighted their crucial contributions in the carcinogenesis (**Figure 3B**). Genomics data of pan-cancer revealed their CNV and SNV landscape, which might be responsible for their expression traits (**Figures 3C–E**). Specifically, SLCO1B3, PAK3, NOX4, MUC4, MMP9, MET, LAMB3, LAMA3, ITGB4, ITGA2, FN1, and EDIL3 genes exhibited obvious SNV traits. Furthermore, almost all of the DE-ARGs exhibited evident mutational patterns in patients diagnosed with SKCM and UCEC (**Figure 3C**). Among the 40 DE-ARGs, the top three genes in the proportion of mutations were MUC4, LAMA3 and FN1, respectively (**Figure 3D**). Additionally, the methylation levels of DE-ARGs showed a significant difference in pan-cancer tissues and para-cancerous tissues (**Figure 3F**). CDH3, EDIL3, PDK4, and PLAT displayed relatively high methylation levels, while SLPI, CEACAM5, PAK3, TRIM31, and MMP13 displayed relatively low methylation levels (**Figure 3F**). Ultimately, the results of ssGSEA uncovered the significant correlation between DE-ARGs and several typical cancer-related pathways (**Figure 3G**). In particular, DE-ARGs were significantly correlated with the activities of typical cancer-related pathways.

### Cluster analysis of 930 patients with PC based on the anoikis scores

**Figure 4:**
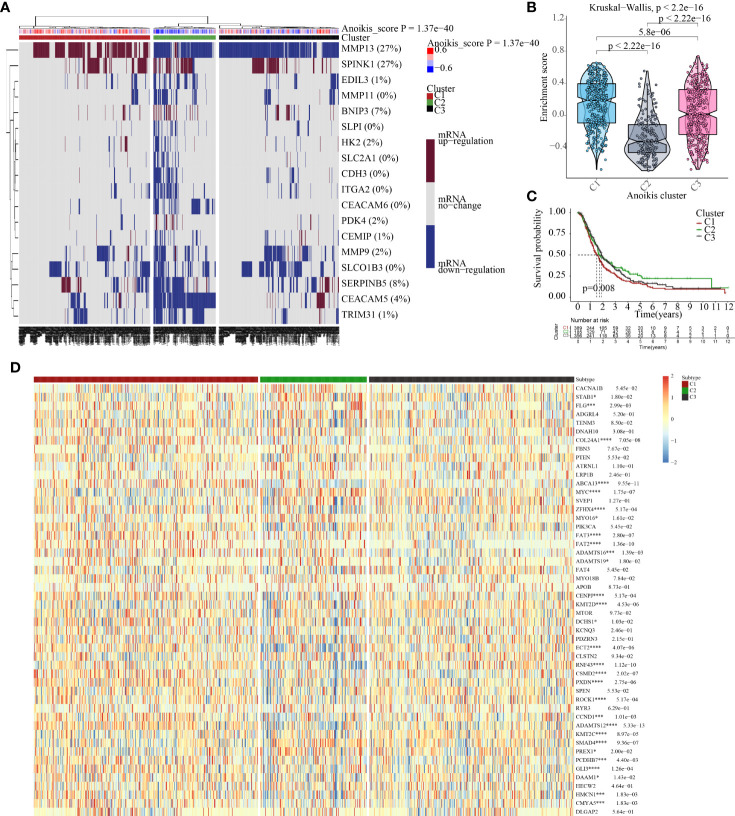
Cluster analysis help identify molecular heterogeneity of patents with PC. **(A)** Cluster analysis based on the anoikis scores obtained from the ssGSEA algorithms. **(B)** Distribution of anoikis enrichment scores among three subtypes (Score: C1 > C3 > C2). **(C)** Cluster-based survival analysis. The overall survival time is C2 > C3 > C1. **(D)** Expression traits of cancer-related genes among three subtypes. *p<0.05, ***p<0.001,****p<0.0001.

**Figure 5:**
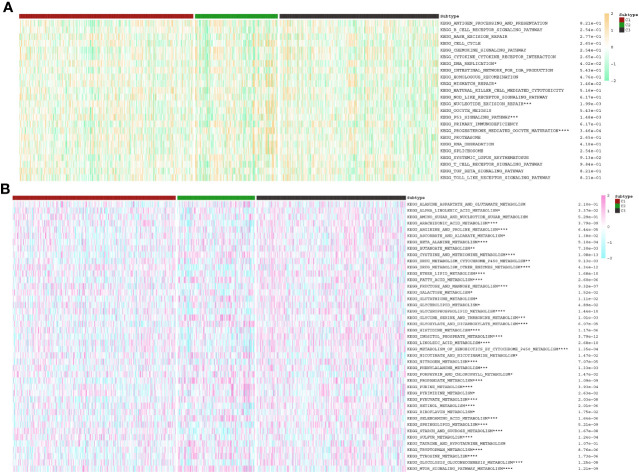
The discrepancies in the activities of **(A)** immune and **(B)** metabolism pathways among three PC clusters. *p<0.05, **p<0.01, ***p<0.001, ****p<0.0001.

Initially, ssGSEA was utilised to compute the anoikis scores of each PC patient. Subsequently, cluster analysis was conducted to classify 930 PC patients into three subtypes, namely C1, C2, and C3 (**Figure 4A**). The anoikis scores among the three subtypes demonstrated a significant difference, with C1 having the highest score, followed by C3, and C2 having the lowest score (**Figure 4B**). More significantly, subtype C1 demonstrated the poorest prognosis while subtype C2 exhibited the most favourable prognosis (**Figure 4C**). Additionally, the C1 subtype was observed to be accompanied by oncogene activation, whereas the C2 subtype was characterised by oncogene inhibition (**Figure 4D**). The aberrant expression of oncogenes may account for the variation in clinical outcomes among PC subtypes. Furthermore, our investigation revealed that immune and metabolic pathways were differentially activated in PC patients with distinct anoikis scores, which is of great significance (**Figures 5A, B**).

### Tumor immune microenvironment analysis

**Figure 6:**
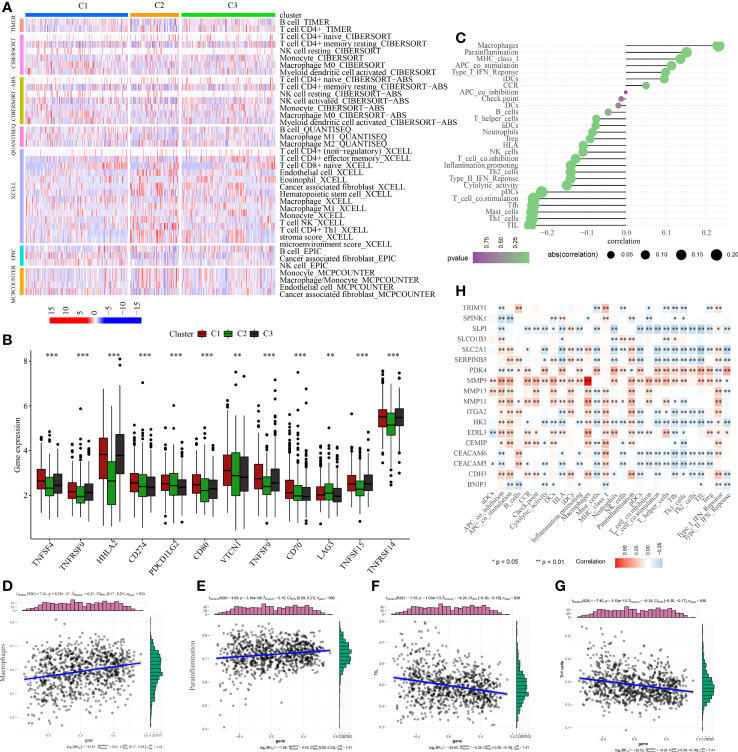
Analysis of tumor immune microenvironment. **(A)** Discrepancies in the immunocyte infiltration among three clusters. **(B)** Discrepancies in the immune checkpoint expression among three clusters. **(C)** Correlation among anoikis scores, immune cell infiltration and immune-related functions. Correlation between anoikis scores and **(D)** macrophages, **(E)** parainflammation, **(F)** TIL, and **(G)** Th1 cells. **(H)** Correlation among DE-ARGs expression and immune cell infiltration and immune-related functions. *p < 0.05; **p < 0.01; ***p < 0.001.

As shown in **Figure 6A**, C2 subtype exhibited a higher proportion of immune cell infiltration, while C1 subtype demonstrated a lower proportion of immune cell infiltration. It is widely acknowledged that immune cells play a crucial role in anti-tumor biological processes. A higher proportion of immune cells often indicate a stronger anticancer activity in the tumor microenvironment, although the regulatory role of ICGs cannot be ignored. Therefore, we conducted additional analysis on the expression distributions of ICGs among the three subtypes. The findings revealed that C1 subtype exhibited higher expression levels of ICGs, whereas C2 subtype demonstrated lower expression levels of ICGs (**Figure 6B**).

To examine the regulatory functions of anoikis in the tumor immune microenvironment, Spearman correlation analysis was conducted to explore the close relationship between anoikis scores and the immune microenvironment (**Figure 6C**). The findings revealed a positive correlation between anoikis scores and macrophage infiltration and parainflammation (**Figures 6D, E**), but a negative correlation with TIL and Th1 cell infiltration (**Figures 6F, G**). Ultimately, we also discovered that the majority of DE-ARGs exhibited significant correlations with immune cell infiltration and immune-related functions (**Figure 6H**). Specifically, MMP9, MMP13, MMP11, and CEMIP were positively associated with the tumor immune microenvironment, while SLPI, SLC2A1, SERPINB5, HK2, CEACAM6, and CEACAM5 were negatively associated with the tumor immune microenvironment.

### Cluster analysis of 226 patients with PNETs based on the anoikis scores

**Figure 7:**
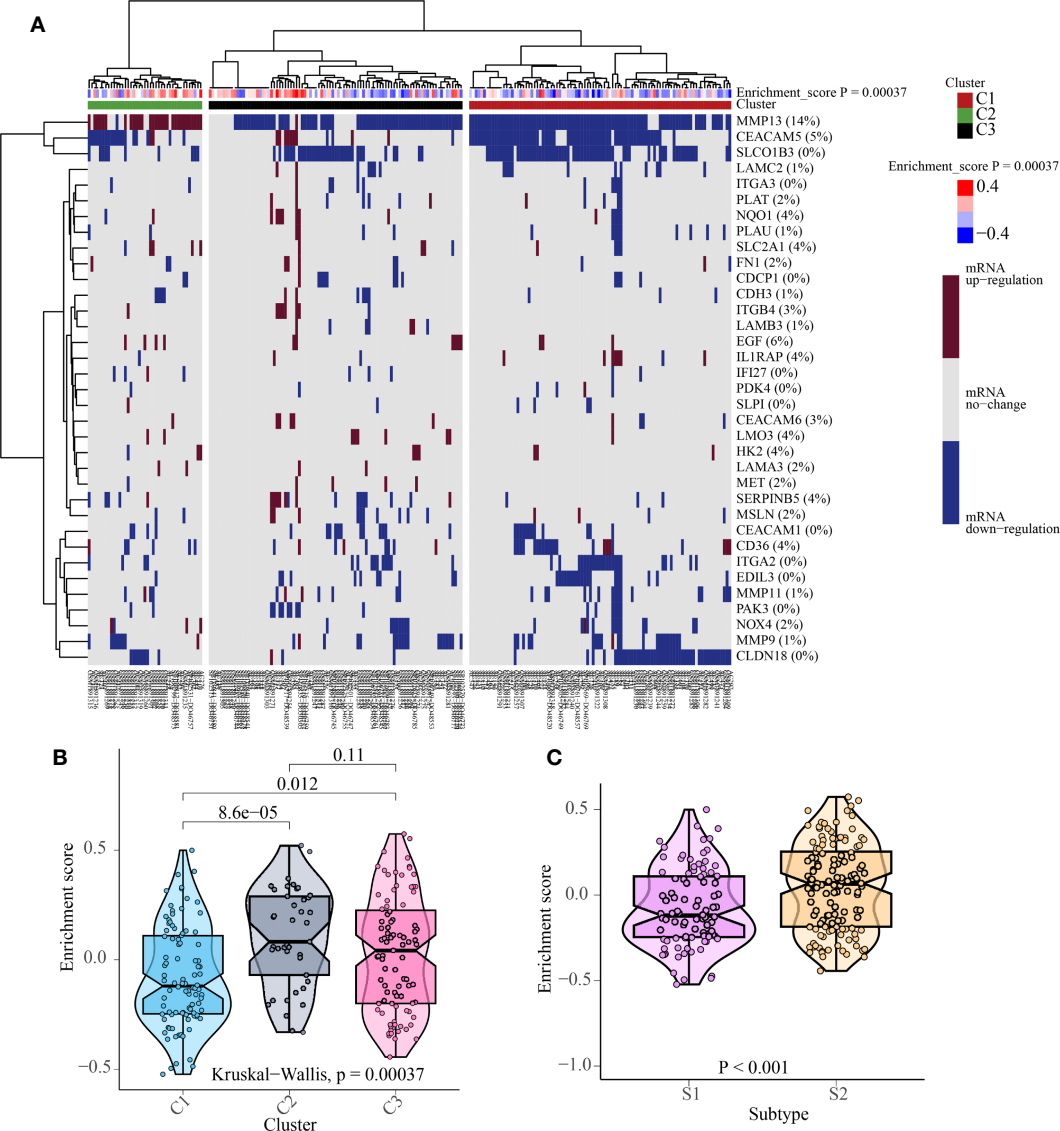
The cluster results of 226 patients with PNETs. **(A)** The unsupervised cluster of 226 patients with PNETs based on anoikis scores. **(B)** The enrichment scores of three clusters of PNETs. **(C)** The enrichment scores of two subtypes of PNETs.

**Figure 8:**
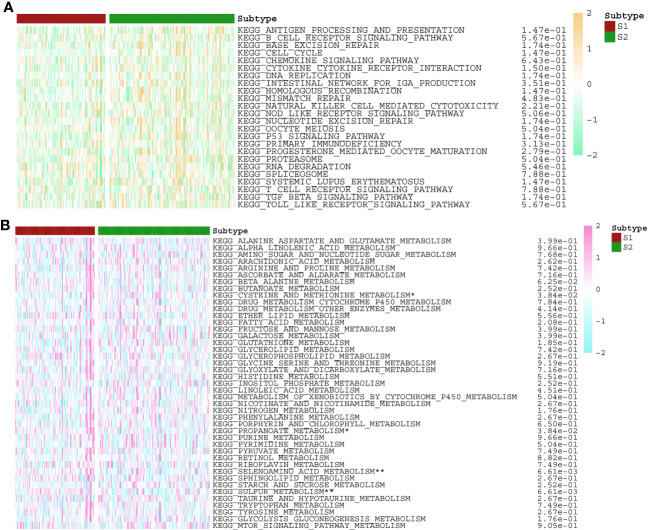
The activities of **(A)** immune and **(B)** metabolism pathways in S1 and S2 subtypes with PNETs. *p < 0.05, **p < 0.01.

According to the enrichment scores of each patient with PNETs, 226 samples were successfully classified into three clusters (**Figure 7A**). The enrichment scores of clusters C2 and C3 were significantly higher than that of cluster C1; however, there was no significant difference between clusters C2 and C3 (**Figure 7B**). Hence, C1 cluster was redefined as S1 subtype with low enrichment scores, while C2 and C3 clusters were merged and redefined as S2 subtype with high enrichment scores (**Figure 7C**). Further investigation was conducted to examine the differences in immune and metabolic characteristics between the two subtypes. Notably, there was no significant variation in the typical immune pathways between the S1 and S2 subtypes (**Figure 8A**). However, the activities of cysteine and methionine metabolism, propanoate metabolism, selenoamino acid metabolism, and sulfur metabolism were found to be significantly different between the S1 and S2 subtypes (**Figure 8B**).

### Identification and verification of a novel ARG-based prognostic model

**Figure 9:**
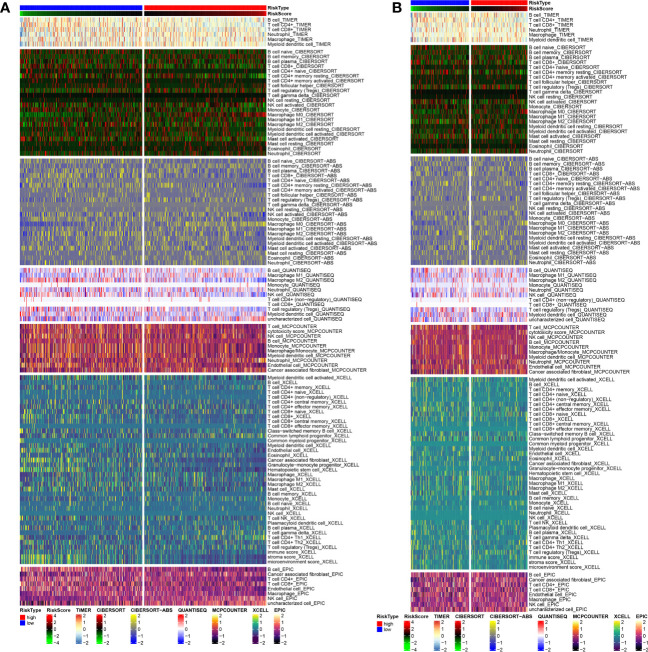
Tumor immune microenvironment analysis in **(A)** training and **(B)** test cohorts.

Taking into account the pathogenic impact of anoikis on PC, we postulated that DE-ARGs could facilitate the development of a new and robust prognostic model. The 40 DE-ARGs were inputted into a LASSO regression model in both the training and test datasets, resulting in the identification of 13 genes (**Figures S1A, B**). The risk score of prognostic model was computed as following: risk score = 0.190969310613421 * HK2 + 0.0519064867507077 * MMP11 + 0.0489408194769506 * MMP9 + (-0.0210122866868812) * CEACAM5 + (-0.0168359640064986) * MMP13 + 0.100798963932116 * BNIP3 + 0.077410041091489 * SLCO1B3 + (-0.0694892084881754) * EDIL3 + 0.059818949860188 * CDH3 + (-0.00847433457654591) * PDK4 + 0.028246530675805 * SERPINB5 + (-0.052088581916376) * CEMIP + 0.0833490721065633 * SLC2A1. The patients in both the training and test cohorts were classified into high-risk and low-risk PC subgroups. In both cohorts, there was a significant survival advantage in the low-risk group compared to the high-risk group (P< 0.05) (**Figures S2A, B**). The risk scores were computed, and the median threshold of the risk score was set at 2.364683 to differentiate between high- and low-risk groups (**Figures S2C, D**). Survival scatter plots of the two cohorts demonstrated a negative association between survival time and the risk score, suggesting that patients in the high-risk group had poorer prognosis (**Figures S2E, F**). The time-dependent ROC curves for overall survival at 1, 3, 5, and 7 years in the training and test groups demonstrated excellent predictive performance using this model (**Figures S2G, H**). Ultimately, we also investigated the differences in ICI between the high- and low-risk subgroups. As shown in **Figures 9A, B**, low-risk PC patients exhibited a higher proportion of ICI than the high-risk subgroup, consistent with the finding that the low-risk subgroup had a significant survival advantage.

### Clinical significances of model genes in PC

**Figure 10:**
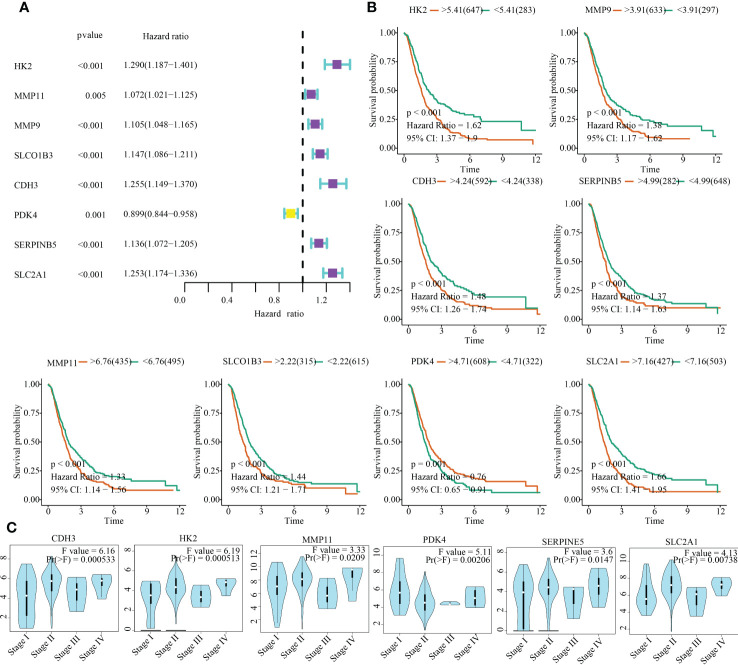
Clinical significances of model genes in PC. **(A)** Uninariate Cox regression analysis. **(B)** Kaplan-Meier survival analysis. **(C)** Correlation between clinical stage and gene expression.

To emphasise the clinical significance of the 13 model genes in PC, we investigated the relationship between these genes and clinical outcomes, as well as clinical stages. The outcomes of univariate Cox regression analysis and KM survival analyses indicated that HK2, MMP11, MMP9, SLCO1B3, CDH3, PDK4, SERPINB5, and SLC2A1 were significantly associated with the survival time of PC patients (**Figures 10A, B**). In addition to PDK4, high expression levels of the other seven genes are unfavourable for the clinical outcomes of PC patients (**Figures 10A, B**). Moreover, HK2, MMP11, CDH3, PDK4, SERPINB5, and SLC2A1 expression were closely associated with tumour stages (**Figure 10C**).

**Figure 11:**
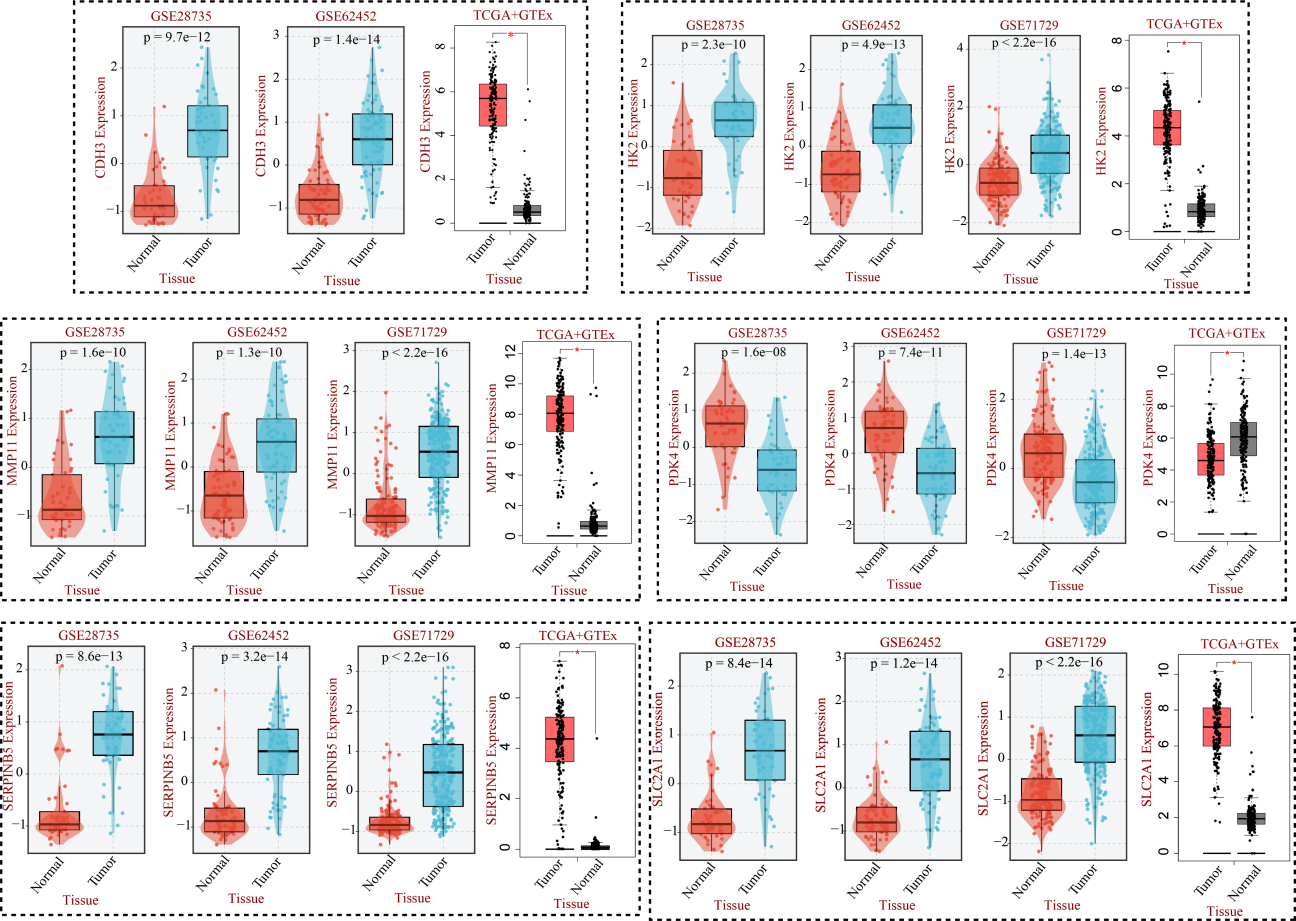
The transcriptomic levels of model genes in tumor and normal tissues. *p < 0.05.

**Figure 12:**
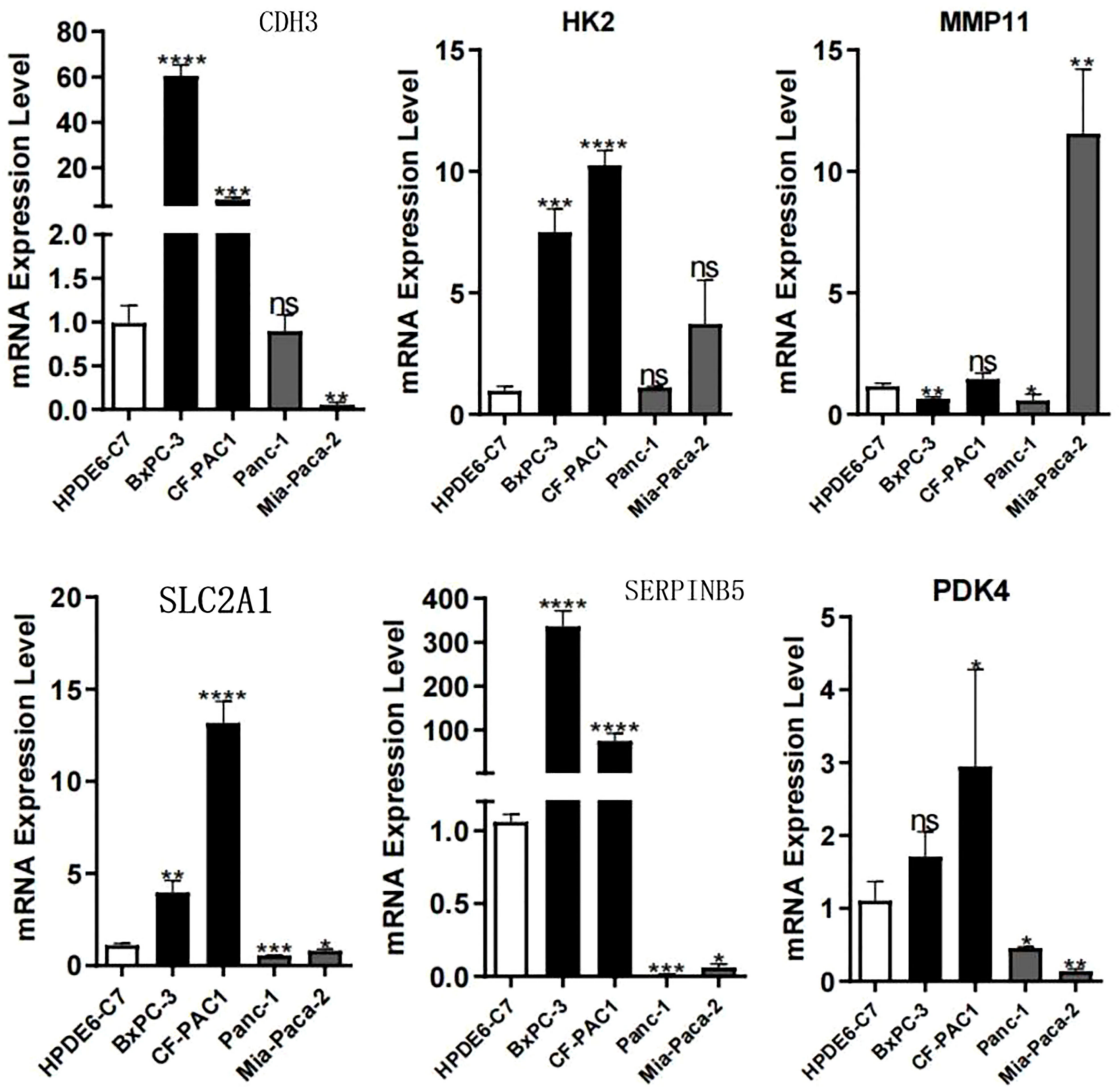
PCR experiments validated the expression levels of model genes. *p < 0.05; **p < 0.01; ***p < 0.001; ****p < 0.0001; ns, no significance.

**Figure 13:**
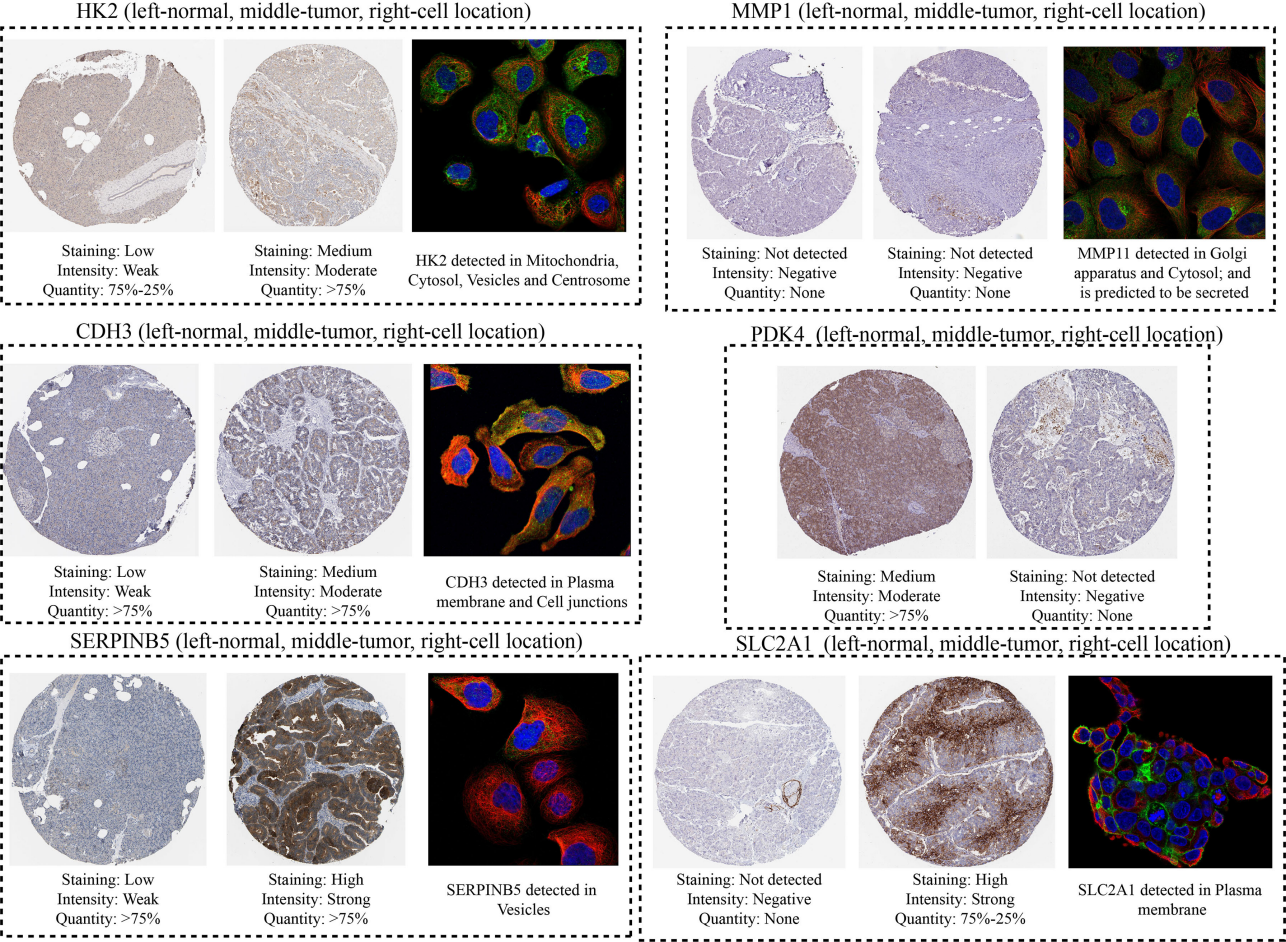
Immunocytochemistry and immunofluorescence results of model genes.

After compiling a series of public PC cohorts, we also observed significant differences in the expression trends of HK2, MMP11, CDH3, PDK4, SERPINB5, and SLC2A1 between PC and para-cancerous tissues (**Figure 11**). It should be noted that HK2, MMP11, CDH3, SERPINB5, and SLC2A1 exhibited increased expression levels in PC tissues, while PDK4 showed decreased expression levels in PC compared to para-cancerous tissues (**Figure 11**). Furthermore, the qPCR results from cell lines confirmed the aforementioned expression trends of HK2, MMP11, CDH3, PDK4, SERPINB5, and SLC2A1 (**Figure 12**). More significantly, the IHC results were in line with the previous transcriptomics findings. The protein expression levels of HK2, CDH3, SERPINB5, and SLC2A1 were significantly higher in PC samples compared to para-cancerous samples. In contrast, the translational level of PDK4 was significantly lower in PC samples (**Figure 13**).

## Discussion

The severity and progression of PC and PNETs pose a challenging clinical problem. Multi-omics has refined our understanding of the rudimentary genetics of PC and PNETs. Although multimodal therapy, including surgery, chemotherapy, radiation, targeted therapy, and immunotherapy, has extended the survival time of pancreatic tumor patients, treatment outcomes remain inadequate. Varied prognoses and clinical responses are observed among individuals with distinct subtypes of PC and PNETs. Prognostic models may accurately identify patients who would benefit from more aggressive treatment, such as extensive surgery, radiation therapy, neoadjuvant chemotherapy, or immunotherapy. Therefore, the development of molecular diagnostic biomarkers and therapeutic targets for PC and PNETs should be given priority.

Anoikis, a specialized kind of programmed cell death, plays a crucial role in body development, tissue homeostasis, disease manifestation, and tumor spread. In-depth research on anoikis has progressively identified the underlying molecular process. Anoikis triggers cell death, integrins sense and transduce extracellular matrix signals, and classical apoptotic pathways regulate cell adherence and survival. Bcl2 and its associated proteins play a significant role in the control of apoptosis, and several protein kinase signal molecules serve as regulatory hubs. Previous researches have highlighted the crucial role of anoikis in multiple human diseases, such as cancers.

Firstly, a pan-cancer analysis summarised and emphasised the essential role of ARGs in the onset and development of cancers. Significant differences in expression of certain ARGs were detected between cancers and para-cancerous tissues. These DE-ARGs were also closely associated with the clinical prognosis of patients with cancer, particularly PC. The aberrant expression patterns may be caused by genomic alterations. Therefore, we investigated the CNV and SNV patterns of DE-ARGs in pan-cancer, which further validated the above aberrant expression patterns. Additionally, we explored the methylation levels and pathway regulation relationship of DE-ARGs in pan-cancer. Most ARGs acted as high-methylation genes in PC. Furthermore, the tumour necrosis factor signalling, interferon signalling, inflammatory signalling, endothelial-to-mesenchymal transition signalling, and IL-6/JAK/STAT3 signalling pathways exhibited obvious correlations with the anoikis pathways in PC. Overall, this research was the first to systematically elaborate on the cancer landscape of anoikis, providing a foundation for future studies.

Bioinformatics technology helped to establish molecular classifiers associated with anoikis for patients with PC and PNETs. The classifier successfully stratified a total of 930 patients with PC into three clusters. Significant differences were observed in the activities of anoikis among different subtypes. Patients with high anoikis scores (C1 cluster) had worse clinical outcomes, while those with low anoikis scores (C2 cluster) had favorable prognoses. Moreover, the expression of most oncogenes varied among the three clusters. Specifically, CMYA5, HMCN1, GLI3, PCDHB7, ADAMTS12, CCND1, ROCK1, CSMD2, RNF43, ECT2, CENPJ, FAT3, ZFHX4, ABCA13, and COL24A1 exhibited significant overexpression trends in the C1 cluster.

To investigate the potential mechanisms underlying clinical outcome differences among patients with distinct anoikis scores, we conducted an intensive analysis of the components of the immune microenvironment and expression of ICGs. Increasing evidence suggests that the immunocompetent cell response plays a crucial role in anti-tumour processes. The C2 subtype was associated with a higher proportion of many anti-tumour immune cells and lower expression levels of ICGs. Previous research has demonstrated a correlation between tumour-infiltrating B lymphocytes and favourable prognoses in cancer patients (27–29). The potential mechanisms underlying B-cell-mediated antitumor immunity may involve the secretion of effector cytokines, such as IFN-γ, by B cells, which can polarise T cells towards a Th1 or Th2 response or enhance T-cell responses through their antigen-presenting cell function (30). This distinctive ability of B cells to directly induce cytotoxicity in cancer is demonstrated by CpG-activated B cells, which can eliminate tumour cells through TRAIL/Apo-2L-dependent pathways (31). Similarly, the C2 subtype with favourable prognoses exhibited higher infiltration of B cells. It has been reported that NK cells recognized most tumor cells through two mechanisms: “missing-self recognition” and “stress-induced recognition” (32–34). After recognition, NK cells primarily exert anti-tumor effects through both direct and indirect pathways (35). Additionally, our findings revealed a higher proportion of NK cells in the C2 subtype. Overall, the dysregulation of the immune microenvironment among different anoikis subtypes may account for the differences in clinical outcomes.

In addition, we also explored the potential regulatory association between ARGs and ICI. Anoikis scores were positively correlated with macrophage levels and para-inflammation, and negatively correlated with TIL and Th1 levels, which were consistent with our previous cluster results. Patients with low anoikis scores (i.e. C2 subtype) had a higher infiltration level of TIL and CD4+T cells. This strong anti-tumor immune response might partly explain why the prognosis of these patients was relatively good. We then systematically investigated the correlation between each ARG and ICI. Interestingly, different genes possessed varying immunomodulatory properties. PDK4, MMP9, MMP13, MMP11, and EDIL3 were positively correlated with ICI and immune-related functions, while SLPI, SLC2A1, SERPINB5, HK2, CEACAM6, and CEACAM5 were negatively correlated with ICI and immune-related functions.

Subsequently, we classified a total of 226 PNET patients into three clusters; however, there was no significant difference between C2 and C3 PNET patients. Therefore, we combined C2 and C3 PNET patients into one subtype. As we did not have follow-up information, we were unable to compare the survival time of patients with different PNET subtypes. While there were no significant differences in immune pathways among different PNET subtypes, differences in several metabolic pathways were notable.

Despite the fact that molecular typing is tremendously important for functional mining of anoikis, we must acknowledge that clustering is, to some extent, a black box. It cannot precisely predict the anoikis scores and clinical outcomes for individual patients. Therefore, we have developed a unique and robust prognostic model related to anoikis using the LASSO regression technique. This model includes 13 genes associated with anoikis, including HK2, MMP11, MMP9, CEACAM5, MMP13, BNIP3, SLCO1B3, EDIL3, CDH3, PDK4, SERPINB5, CEMIP, and SLC2A1. There was a significant difference in survival outcomes between high-risk and low-risk pancreatic cancer patients in both the training and validation cohorts. More importantly, ROC curves further validated the prediction accuracy of the model and demonstrated its ability to predict the survival outcome of 930 patients with pancreatic cancer, which could have wide applications in the future and provide a reference value for individual patient intervention.

Hexokinase 2 (HK2) catalyzes the phosphorylation of glucose, a step required for glucose metabolism (36, 37). Anderson et al. have reported that HK2 had the potential to enhance tumor proliferation, growth, invasion, and metastasis *via* regulation of lactate metabolism in PC (38). In individuals with PC, HK2 also prevented cell apoptosis mediated by gemcitabine through voltage-dependent anion channel (39). Remodeling of the extracellular matrix (ECM) by matrix metalloproteinases (MMPs) was a crucial stage in the invasion and metastasis of solid malignant tumors as it enabled tumor cells to modify ECM components and release cytokines, thus promoting protease-dependent tumor progression (40). Cell adhesion, intracellular and intercellular signal transduction, cancer development, inflammation, angiogenesis, and metastasis are just a few of the activities of carcinoembryonic antigen-related cell adhesion molecules (CEACAMs) in complex biological processes. CEACAM5 is now considered a reliable clinical biomarker and a promising therapeutic target for melanoma, lung cancer, colorectal cancer, and pancreatic cancer (41).

However, there are some limitations associated with our research. Our signature was constructed using retrospective data from public datasets. To further establish the predictive significance of our prognostic signature, extensive prospective clinical research is necessary. Furthermore, as the signature was developed using bioinformatics research, additional fundamental research is required to validate our findings. Despite these limitations, our study still holds unique clinical significance. The pan-cancer comprehensive analysis of anoikis is particularly useful for the advancement of further fundamental research in the future. The molecular classifier and prognostic model based on anoikis score aid in identifying the inherent heterogeneity of pancreatic cancer patients, thus promoting the development of personalised intervention therapy for tumors.

## Conclusion

This is the first study to systematically investigate anoikis in pan-cancer, categorize patients with PC and PNETs into unique molecular subtypes according to their levels of anoikis, and create a dependable predictive model for PC based on anoikis. The functional status, tumor immune microenvironment, and clinical outcomes of patients with PC displayed considerable diversity. The survival rate of PC patients could be accurately anticipated by the risk model based on anoikis. Our findings hold the potential to enhance anoikis research and the targeted therapy of patients with pancreatic tumors.

## Data availability statement

The original contributions presented in the study are included in the article/**Supplementary Material**. Further inquiries can be directed to the corresponding authors.

## Author contributions

NL and ZW: The authors are responsible for the study design, data collection, data analysis, writing the manuscript, making the figures. XJ, KW, ZQ: The author is responsible for data collection, data analysis, revising the manuscript, making the figures. DC, ZS: The authors contribute to the study design, data collection, and writing the paper. JJ, YC, and CW: The authors supervised the project, designed this study, revised the manuscript. All authors contributed to the article and approved the submitted version.

## Supplementary material

Supplementary Figure 1 The processing of LASSO algorithms.

Supplementary Figure 2 Development and validation of the anoikis-based prognostic model. Survival curves of the anoikis-based prognostic model in **(A)** training and **(B)** test cohorts. Division of high-risk and low-risk groups in **(C)** training and **(D)** test cohorts. Distribution of survive time and risk scores in **(E)** training and **(F)** test cohorts. ROC curves of the anoikis-based prognostic model in **(G)** training and **(H)** test cohorts.
