# Supplementary figures and images for: Characterization of anoikis-based molecular heterogeneity in pancreatic cancer and pancreatic neuroendocrine tumor and its association with tumor immune microenvironment and metabolic remodeling
Source: Front Endocrinol (Lausanne). 2023 May 10;14:1153909. doi: 10.3389/fendo.2023.1153909 (PMC10206226; doi:10.3389/fendo.2023.1153909)

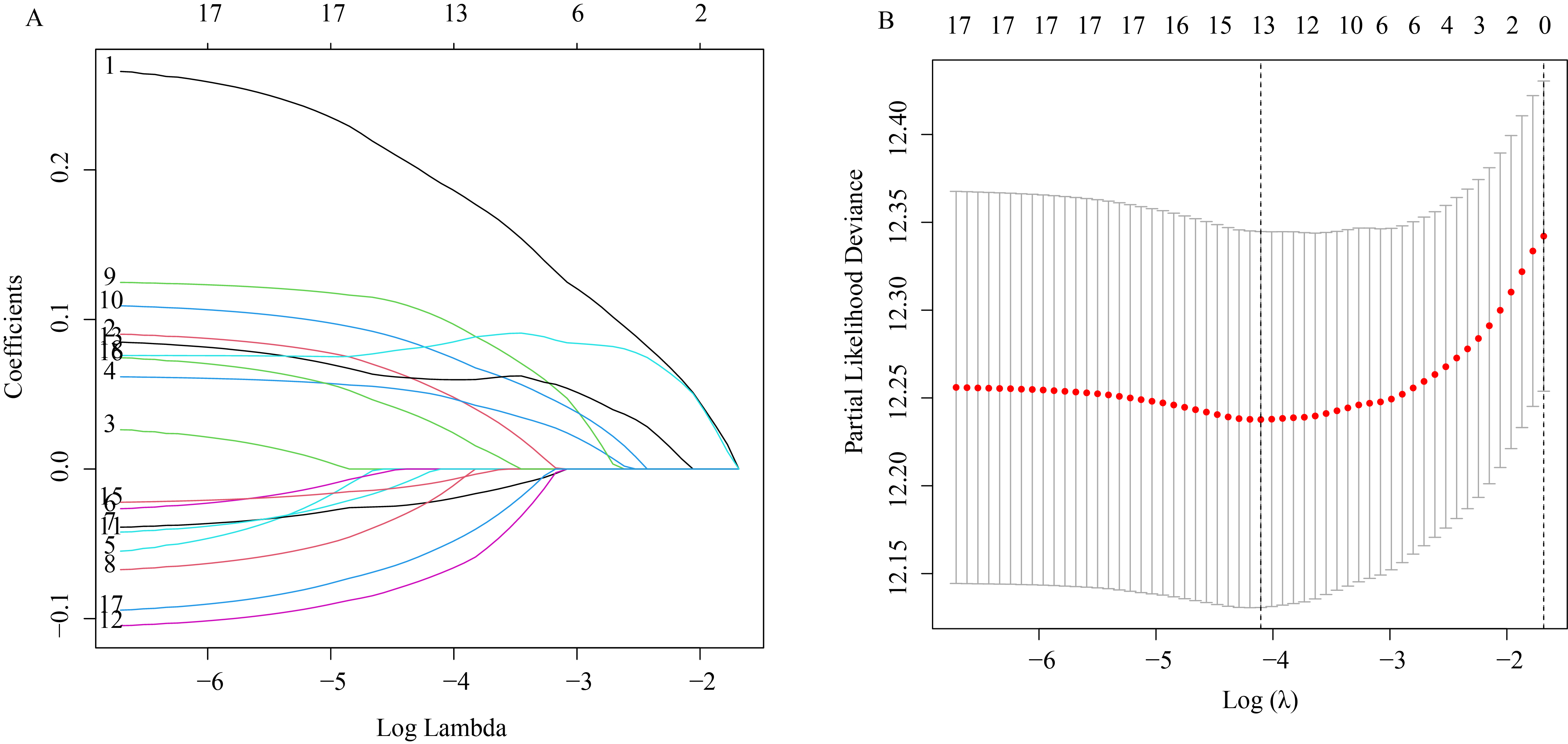

Supplement: Supplementary Figure 1 — The processing of LASSO algorithms. [file Image_1.jpeg]

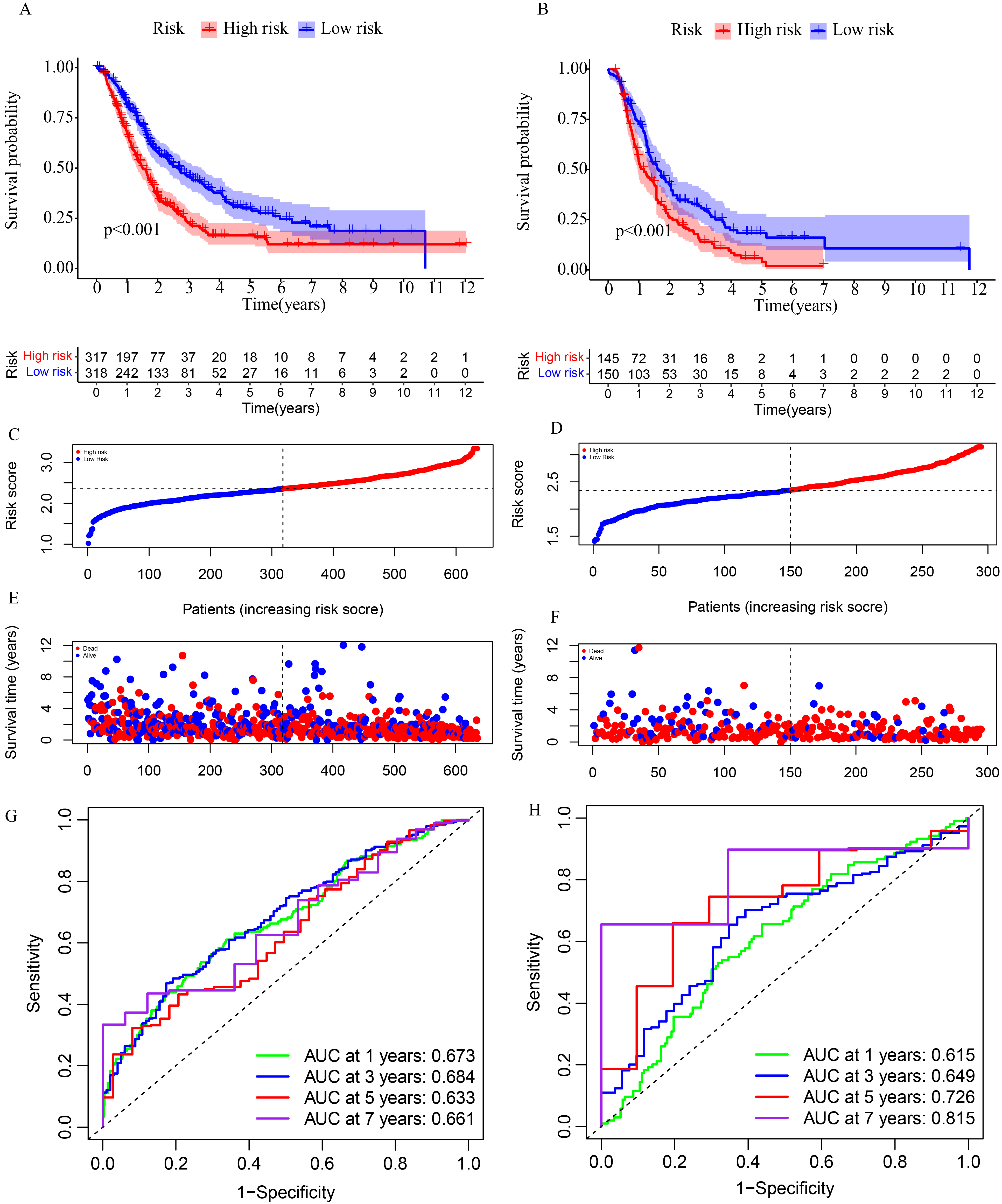

Supplement: Supplementary Figure 2 — Development and validation of the anoikis-based prognostic model. Survival curves of the anoikis-based prognostic model in (A) training and (B) test cohorts. Division of high-risk and low-risk groups in (C) training and (D) test cohorts. Distribution of survive time and risk scores in (E) training and (F) test cohorts. ROC curves of the anoikis-based prognostic model in (G) training and (H) test cohorts. [file Image_2.jpeg]
